# Covalently Linked 2D-Co_3_O_4_/GO Heterostructures: Catalytic and Electrochemical Properties

**DOI:** 10.1021/acs.langmuir.4c02235

**Published:** 2024-10-03

**Authors:** Jéssica E. S. Fonsaca, Carlos Eduardo Lima, Kevin Stefan Boszko Martins, Sergio H. Domingues, Christiano J. S. de Matos

**Affiliations:** † School of Engineering, Mackenzie Presbyterian University, Sao Paulo 01302-907, Brazil; ‡ MackGraphe, Mackenzie Presbyterian Institute, Sao Paulo 01302-907, Brazil

## Abstract

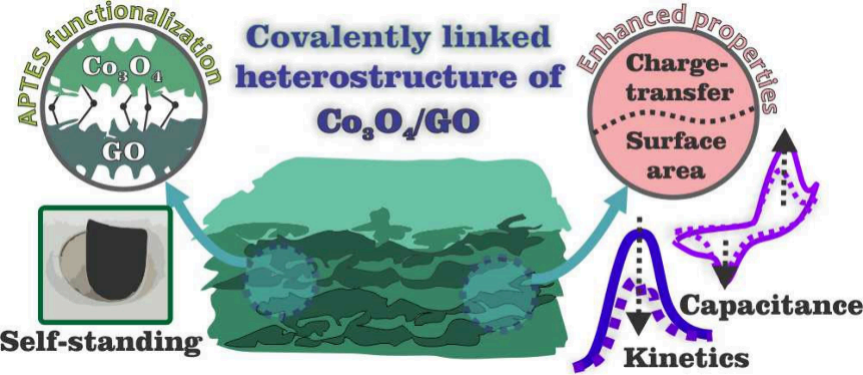

Covalently cross-linked 2D heterostructures may represent a ground-breaking approach to creating materials with multifunctionalities. To date, however, this field still remains relatively unexplored. In the present work, Co_3_O_4_/GO covalently linked heterostructures (Co_3_O_4_/GO-CL) were produced using 2D-Co_3_O_4_ functionalized with (3-aminopropyl)triethoxysilane (APTES) to react with the carboxyl groups of graphene oxide (GO). The surface and interface properties of the final material were assessed through electrochemical and catalytic studies. We found that the covalent bonds lead to a self-standing and ordered final structure, not observed for the noncovalent material (Co_3_O_4_/GO-nCL), also produced for comparison. The catalytic activity of Co_3_O_4_/GO-CL over the degradation of Rhodamine 6G showed great performance and the possibility of recycling the catalyst. Electrochemical evaluation stated higher specific capacitance for the covalently bonded material (468 F g^–1^ against 110 F g^–1^). Overall, results showed that the covalent bonds may be improving charge-transfer and interfacial area features, thus leading to enhanced catalytic and electrochemical performances.

## Introduction

The vertical stacking of two-dimensional (2D) materials has become an appealing idea for reaching, in one single platform, the best of what different 2D crystals have to offer. These 2D homo or heterostructures have been highly explored and materials such as graphene-BN,^1^ graphene-MoS_2_,^2^ MoS_2_–WSe_2_,^3^ etc. have shown fascinating properties. More commonly, these structures have been produced through the stacking of one layer on top of the other, which are kept together by van der Waals (vdW) interactions.^4^ This fabrication method, however, is laborious since it requires manual transferring steps using supporting polymers.^5^ Also, as a result, organic/polymer residues may remain trapped between the layers.^6^ Besides that, the lack of control over the distance and chemical nature of the interfaces limits the achievable structures.^7^

A way out of these obstacles may be obtained by the covalent bonding between the 2D structures, intermediated by molecules leading to cross-linked layered structures^8^ that compose a whole new class of 2D heterostructures, which is still poorly explored in the literature. Heterogeneous and homogeneous cross-linked structures have been mainly synthesized through the Sonogashira and 1-ethyl-3-(3-(dimethylamino)propyl)carbodiimide hydrochloride (EDC) couplings.^9,10^ Thus, the formation of sandwich-like well-known structures, mediated by strategic functional groups to covalently link the layers, may enable control of chemical reactivity of the stackings, opening a range of applications for these platforms.

These structures have specially been shown to present prominent performance when applied as electrocatalysts,^10−13^ which is mainly related to enhanced charge-transfer interactions and superior surface area. Moreover, they have displayed excellent stability when submitted to thousands of cycles.^13^ Rao et al.^10^ have shown that these features can be dictated by the distance and the nature of the interactions between the layers. When covalently bonded graphene–borocarbonitride heterostructures were employed as electrocatalysts for hydrogen evolution reaction (HER),^14^ the best catalyst turned out to be connected by shorter linkers, rather than longer ones. The highest efficiency is attributed to more effective charge transfer between the sheets. When evaluated in the adsorption of CO_2_ and H_2_, the optimization of the linkers has also impacted on the performance of the final material. Rao et al.^15^ demonstrated that by tuning the specific area of 3D frameworks of reduced graphene oxide (rGO) via the choice of organic linkers, the homostructure adsorption capacity could be optimized. Other structures such as MoS_2_–rGO,^16^ MoS_2_–MoS_2_,^16^ MoS_2_–boron carbonitride,^17^ and C_3_N_4_ with both MoS_2_ and rGO,^12^ have also shown enhanced properties for different applications.^8^

Definitely, the interfaces between 2D layers are confined spaces where atoms and molecules are prone to intercalate and induce unknown chemical processes (e.g., catalytic and/or electrochemical reactions).^18^ Encouraged by the novel properties and opportunities offered by these assemblies and the fact that there is plenty of space for new chemistry to be explored in this field, this approach may be extended to various types of 2D nanomaterials and designed according to the targeted application. Here, we are particularly interested in graphene oxide (GO) or reduced graphene oxide (rGO) and two-dimensional (2D) Co_3_O_4_ hybrids, which are known to display interesting performance in catalytic^19,20^ and electrochemical^21−23^ activities. Generally, the reported nanocomposites are produced through the random combination of the materials, which are based on noncovalent interactions. Indeed, works reporting on the production of heterostructures of 2D-Co_3_O_4_ covalently bonded to GO sheets, as well as the investigation of their catalytic and electrochemical properties, have not been reported. For this purpose, we have explored the chemistry of (3-aminopropyl)triethoxysilane (APTES) and the EDC chemical coupling to modify the surface of Co_3_O_4_ and GO, respectively, and produce a novel hybrid material.

APTES is an aminosilane that attract special attention because of its reactive amino groups, which are nucleophilic centers that may promote additional derivatization, as well as its impressive water solubility that makes it suitable for modifying a wide variety of surfaces.^24,25^ The most common mechanisms for anchoring APTES on different supports are (i) solvolysis reaction in water-free conditions and (ii) reaction via the hydrolysis of the silane agent.^24^ Oxide surfaces that have hydroxyl groups (−OH), such as Co_3_O_4_,^26^ may easily interact with Si–OH termination and form covalent bonds.^27,28^ In this sense, the amino groups would be available for further functionalization, e.g., specific active sites of graphene oxide.

One standard procedure to immobilize structures with free amino groups is through the preparation of succinimidyl ester (−COOSuc)-terminated surfaces, which react with amino linkers to form strong covalent bonds.^29^ This may be achieved by reacting a surface with bearing carboxylic groups (e.g., graphene oxide) with *N*-hydroxysuccimide (NHS) and EDC, thus promoting the activation of the surface and allowing the reaction with primary amines to be carried out in order to form amide bonds.^29^

With these reaction pathways in mind, a hybrid structure based on the covalent linkage between Co_3_O_4_ and GO has been produced (Co_3_O_4_/GO-CL) and has been fully characterized. Thus, aiming at evaluating the surface and interface properties (e.g., charge-transfer and intercalation capabilities) of this material, catalytic and electrochemical studies were carried out and compared with the behavior of a hybrid material obtained by the simple mixture of the precursors (i.e, noncovalently linked Co_3_O_4_/GO-nCL).

For catalysis purposes, we evaluated the degradation of Rhodamine 6G (R6G) (i.e., reduction in the presence of NaBH_4_), which is a dye commonly discharged into water sources.^30^ In this case, the study was carried out through kinetic measurements monitored by UV-vis spectroscopy. Electrochemical investigations were performed by cyclic voltammetry and impedance spectroscopy analysis with the aim of appraising the potential of the heterostructures as electrodes in energy storage devices (e.g., supercapacitors).^31^

## Experimental Section

### Materials

Cobalt acetate tetrahydrate ((CH_3_COO)_2_Co·4H_2_O), Sigma–Aldrich, 99.9%), hexamethylenetetramine (C_6_H_12_N_4_, Sigma–Aldrich, 99.9%), ethylene glycol (Sigma–Aldrich, 99.9%), (3-aminopropryl)triethoxysilane (APTES, Sigma–Aldrich, 99%), *N*-hydroxysuccinimide (NHS, Sigma–Aldrich, 98%), *N*-(3-(dimethylamino)propyl)-*N*-ethylcarbodiimide hydrochloride (EDC, Sigma–Adrich), Rhodamine 6G (R6G, Exciton), ethanol (Sigma–Aldrich, 99.9%), 1-methyl-2-pyrrolidone (NMP, Sigma–Aldrich, anhydrous, 99.5%), carbon black (CABOT), poly(vinylidene fluoride) (PVDF, Sigma–Aldrich). All reagents were used as received.

### Synthesis of Hybrid Structures

**Figure 1:**
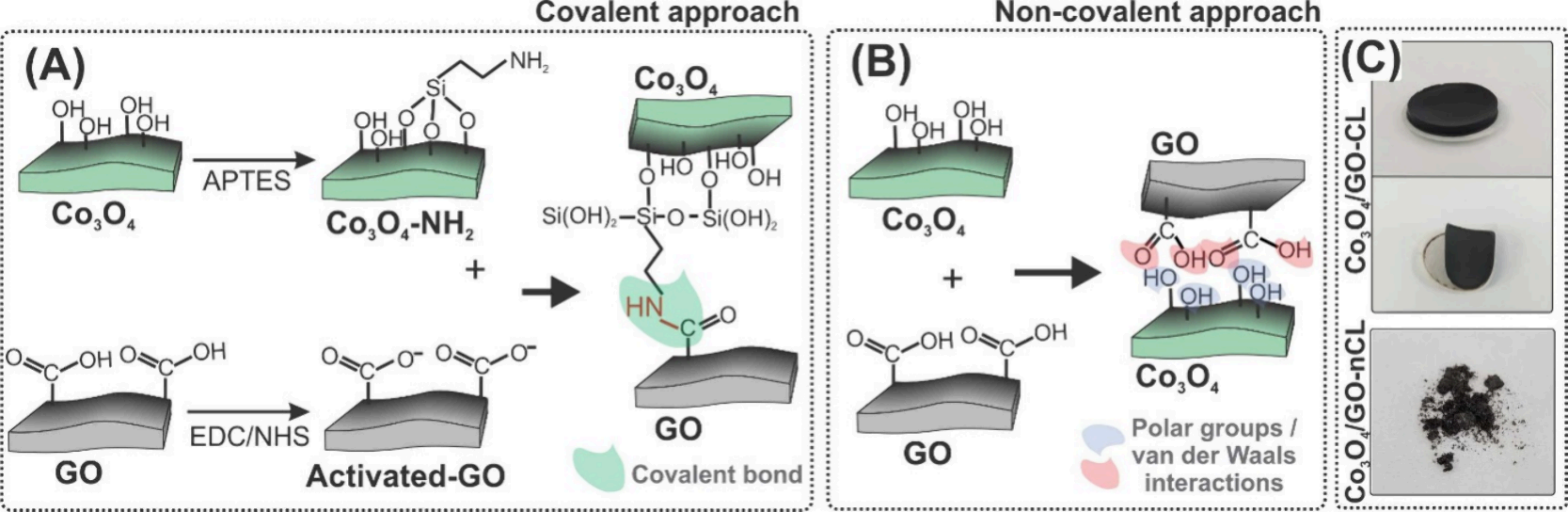
Schematic steps to produce (A) Co_3_O_4_/GO-CL and (B) Co_3_O_4_/GO-nCL synthesis. Digital photographs of (C) Co_3_O_4_/GO-CL (top) and Co_3_O_4_/GO-nCL (bottom).

Details of Co_3_O_4_ and GO synthesis can be found in section S1 of the Supporting Information (SI). The production of the hybrids is illustrated in Figures 1A and 1B, for covalent and noncovalent heterostructures, respectively.

The covalently linked structure was prepared as follows: Co_3_O_4_ was exfoliated in deionized water (0.1 mg mL^–1^) in an ultrasonic bath (37 kHz) for 120 min and the as-produced GO dispersion (1.5 mg mL^–1^) was diluted to the concentration of 0.2 mg mL^–1^. The synthesis of the covalently bonded structure was carried out in two steps. In a first round flask, 2 mL of APTES were added into 38 mL of Co_3_O_4_ dispersion and kept under stirring (1000 rpm) overnight, leading to Co_3_O_4_ modified with APTES (Co_3_O_4_–NH_2_). In a second flask, immersed in an ice bath and containing 40 mL of GO dispersion under stirring (1000 rpm), 8 × 10^–5^ mol of EDC and 7.37 × 10^–5^ mol of NHS were added. After 2 h of reaction, the Co_3_O_4_–NH_2_ mixture was added and left stirring overnight. The resulting material (Co_3_O_4_/GO-CL), was filtered in an acetate membrane under vacuum and dried at 60 °C for 30 min. The resulting Co_3_O_4_/GO-CL leads to a 3D self-standing material that naturally detaches from the membrane, as exhibited in Figure 1C (top).

For the synthesis of the structure without covalent bonds (Co_3_O_4_/GO-nCL, Figure 1B), the methodology was carried out using the same steps and conditions mentioned above but in the absence of the functionalization reagents (APTES, EDC, NHS). The Co_3_O_4_/GO-nCL product, as depicted in Figure 1C (bottom), is obtained as a powder.

### Characterization

A confocal Raman spectrometer (WITec Alpha 300R) was used in the laser line of 532 nm (50× objective lens and 600 g/mm grating) for the acquisition of Raman spectra (power = 0.2 mW, integration time = 1 s, and 20 accumulations). UV-vis spectra were obtained in a UV-vis-NIR spectrophotometer (Shimadzu, Model UV-3600i Plus). A Rigaku Miniflex II system was employed for obtaining X-ray diffraction (XRD) profiles (Cu Kα (λ = 1.5406 Å)). Morphological characterization was carried out through scanning electron microscopy (SEM) in a microscope (JEOL, Model JSM-7800F) with a lower electron detector (LED) under 10 kV. A Fourier transform infrared (FT-IR) spectrometer (Bruker, Vertex 70) was employed for obtaining single spectra of the samples in the infrared region (4000–700 cm^–1^), using a ZnSe window as the substrate. For spectral mapping, the attenuated total reflectance mode was carried out using the Hyperion Microscope (Bruker) coupled to the FT-IR spectrometer. Data were analyzed by using OPUS software. Contact angle analyses were carried out in a goniometer (Kruss, Model DAS-100) by the sessile drop method using deionized water at a temperature of 20 °C directly dropped over the films after filtration. XPS analyses were performed by a Thermo Scientific Model K-Alpha spectrometer using monochromatized Al Kα (*h*ν = 1486.6 eV) excitation radiation with an X-ray spot size of 300 μm. The survey spectrum was obtained with 10 scans, a constant pass of 200 eV, and an energy step size of 1.0 eV. The high-resolution spectra were obtained with 10 scans, a constant pass energy 50 eV, and an energy step size of 0.1 eV.

### Kinetic Measurements

Catalytic efficiency was evaluated through UV-vis spectroscopy in the 200–600 nm range (2 nm step). The reactions were monitored at 525 nm by the degradation of R6G. For this purpose, 0.5 mg of the hybrid materials was added in a 3 mL quartz cuvette containing an aqueous solution of 5.2 × 10^–5^ mol L^–1^ of R6G. Reactions were initiated with the addition of 1.32 × 10^–5^ mol of NaBH_4_. The mixture was stirred (600 rpm) and controlled temperature (20 °C). Absorbance monitoring was carried out by interrupting the stirring at specific times and taking the cuvette to the spectrometer for measurement. Absorbance versus time curves were obtained and first-order rate constants (*k*_obs_) were calculated using an iterative least-squares software program. The coefficient of determination (*R*^2^) values for all analyses were >0.99.

### Electrochemical Measurements

Prior to electrochemical testing, working electrodes were prepared with a mass proportion of 8:1:1 (active material:carbon black:PVDF) in NMP to make a homogeneous slurry and deposited in nickel foam substrates. The prepared working electrodes were then dried under vacuum at 70 °C overnight and pressed at 10 MPa to improve adherence. Working electrodes had ∼3.0 mg of active materials and a coverage area of 0.4 cm^2^. The exact amount was obtained for each electrode by weighing the foam substrates before and after slurry deposition. Through the mass difference, the mass of the active material could be calculated.

Electrochemical measurements were performed in a Metrohm Autolab PGSTAT302N conventional three-electrode system: a reference electrode (saturated Ag/AgCl), a counter electrode (Pt wire), and a working electrode (as described above). The electrolyte used was 2.0 M KOH. Cyclic voltammetry was evaluated at different scan rates between 0.0 V to +0.6 V of potential window, and electrochemical impedance spectroscopy (EIS) was performed at open-circuit potential with an amplitude of 5 mV from 10^5^ Hz to 10^–2^ Hz. EIS data was analyzed with the NOVA 2.1 software program.

## Results and Discussion

### Materials Characterization

**Figure 2:**
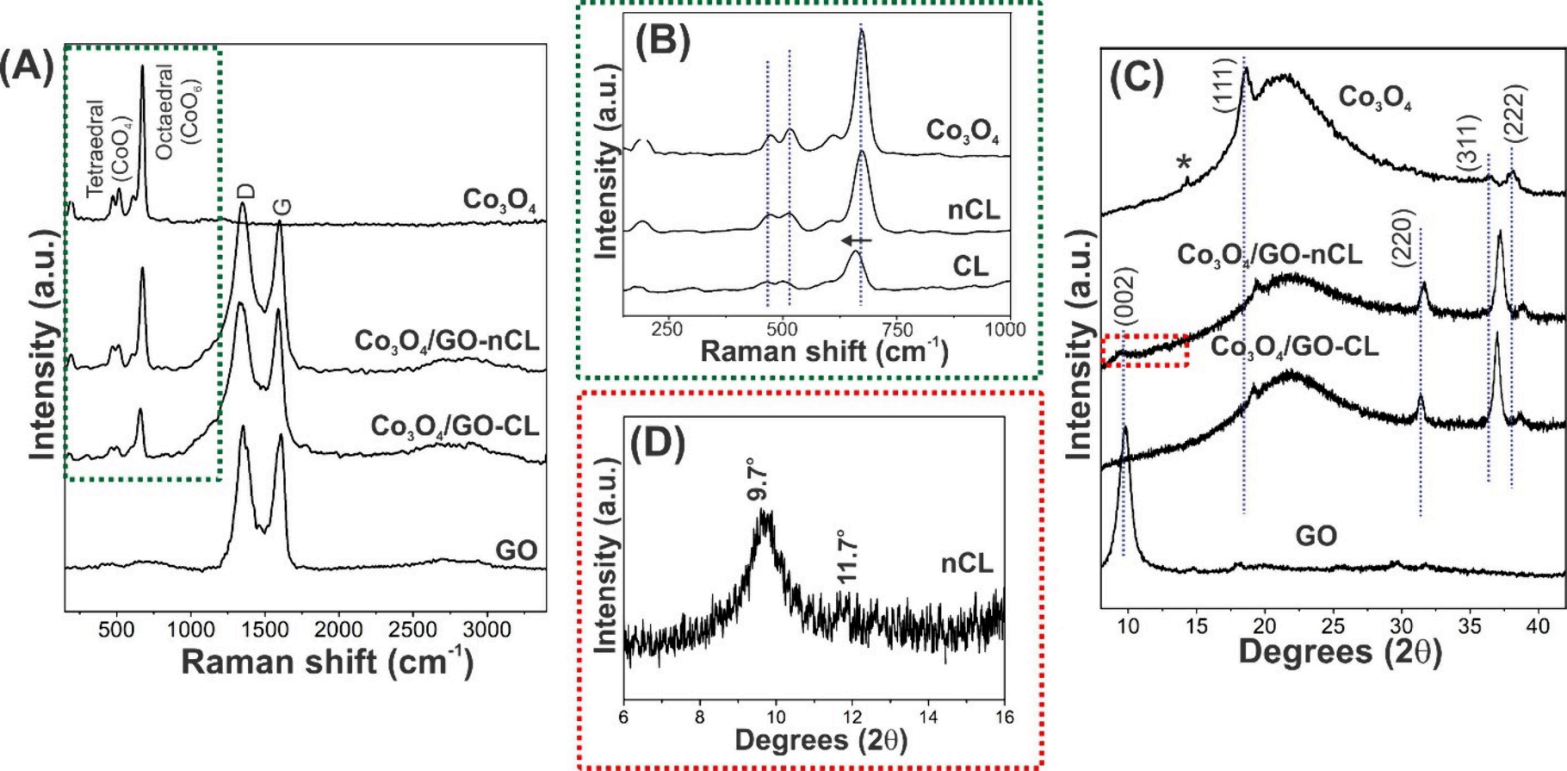
(A, B) Raman spectra (λ = 532 nm) and (C, D) X-ray diffractograms of pure components 2D-Co_3_O_4_ and GO, and the hybrid structures Co_3_O_4_/GO-CL and Co_3_O_4_/GO-nCL. Panels (B) and (D) correspond to expanded sections of the marked areas in panels (A) and (C), respectively.

Structural information about the heterostructures, in comparison to the bare components, was first obtained through Raman spectroscopy and XRD analysis and is presented in Figure 2. Overall, Raman data (Figure 2A) shows that separate samples of 2D-Co_3_O_4_ and GO present the typical profiles of each component: (i) exfoliated Co_3_O_4_ displays bands at 192, 473, 523, and 613 cm^–1^, corresponding to F_2g_, E_1g_, F_2g_, and F_2g_ modes of tetrahedral sites, and at 677 cm^–1^, related to the A_1g_ mode of the octahedral sites of the oxide,^32^ and (ii) GO exhibits D and G bands at 1352 and 1600 cm^–1^, respectively,^33^ which are common fingerprints of graphene derivatives. All bands are concomitantly found in the heterostructures spectra, attesting to the synthesis of hybrid structures in both cases (cross-linked and non-cross-linked). By comparing in more detail, the Raman spectrum of pure 2D-Co_3_O_4_ with those of the hybrids (see Figure 2B), it is possible to note that the noncovalent Co_3_O_4_/GO does not indicate any considerable change. In contrast, the spectrum of Co_3_O_4_/GO-CL clearly shows the shift of the A_1g_ mode to lower frequencies (∼16 cm^–1^, see the arrow in Figure 2B). This is consistent with the creation of a more-defective structure,^34^ which could be related to the insertion of new functional groups onto 2D-Co_3_O_4_ surface through the covalent bonds.

Figure 2C shows the XRD patterns of 2D-Co_3_O_4_, GO, Co_3_O_4_/GO-CL, and Co_3_O_4_/GO-nCL. The diffractogram of the pure Co_3_O_4_ exhibits the characteristic peaks of spinel Co_3_O_4_ phase,^35,36^ at ∼18.5°, 36.5° and 38°, consistent with the (111), (311) and (222) planes. Generally, these peaks are also observed in both heterostructures. In all cases, however, they are shifted to higher 2θ angles, indicating a lattice contraction^37^ or strain^38^ of the components. This may be a result of the introduction of defects in Co_3_O_4_, because of van der Waals (vdW) interactions or covalent bonds, which are necessary for the formation of the heterostructures.

Regarding interlayer spaces, a peak at 14° (denoted by an asterisk in Figure 2C) indicate a distance of 0.63 nm for pure exfoliated Co_3_O_4,_ while pure GO presents the most prominent peak at 9.8°, related to the (002) plane^39^ and an interlayer distance of 0.92 nm.

It is interesting to note that these peaks, which are related to interlayer spaces, do not appear in the Co_3_O_4_/GO-CL diffractogram. The absence of these reflections suggest that the cross-linked composite contains monolayers of Co_3_O_4_ and GO in the new structure.^12^ In contrast, for Co_3_O_4_/GO-nCL, these peaks are found to be shifted to lower 2θ (see Figure 2D), which implies that the layers are stacked in a layer-by-layer mode with an increased interlayer distance due to the insertion of new sheets in this arrangement.

Curiously, the diffractogram of the hybrids present a peak at ∼31.5° that refers to the (220) plane of spinel Co_3_O_4_,^36^ which, however, does not appear in the diffractogram of pure exfoliated Co_3_O_4_. Somehow, the exposure of this plane has only been favored after the formation of both heterostructures.

**Figure 3:**
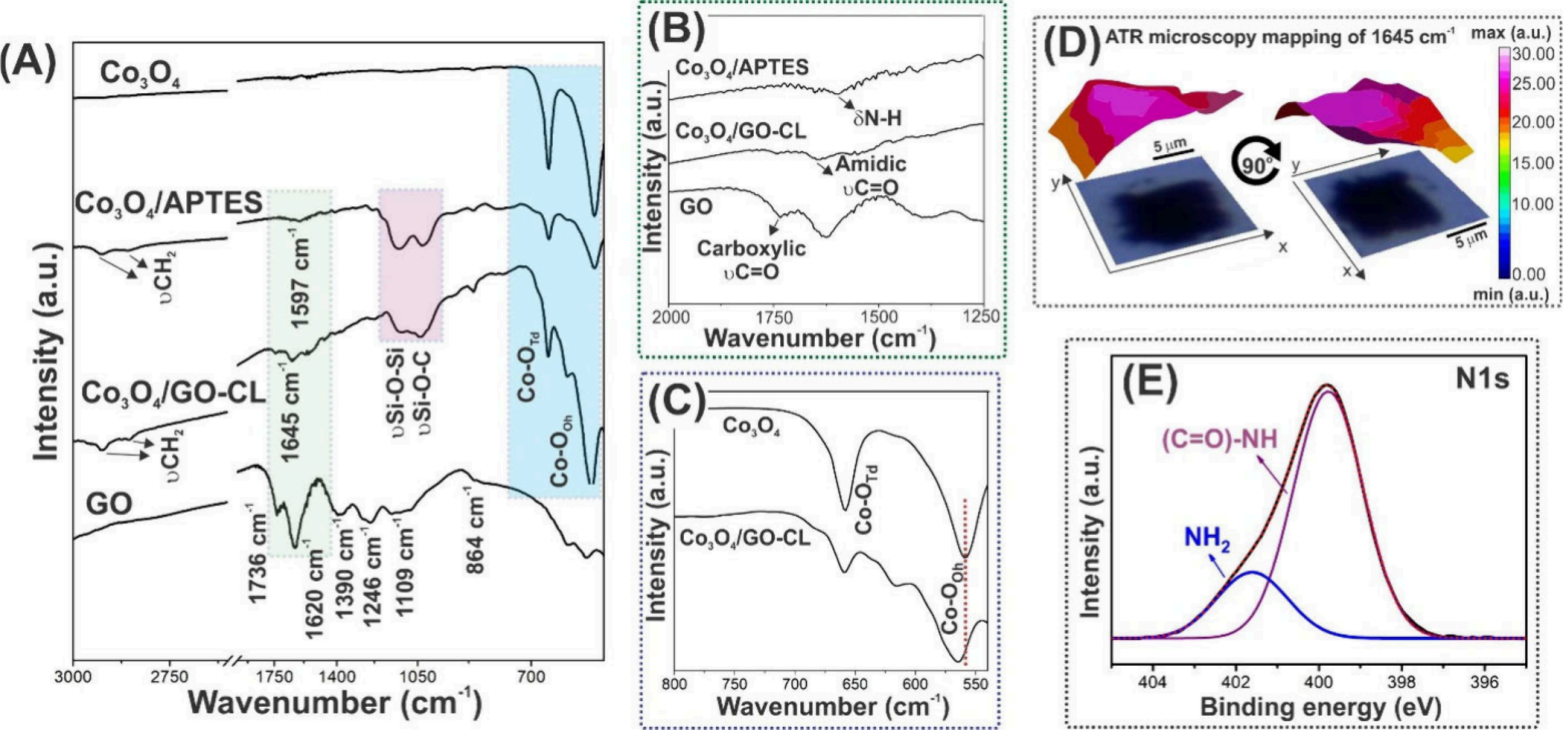
(A–C) FTIR spectra of Co_3_O_4_, Co_3_O_4_/APTES, and Co_3_O_4_/GO-CL and GO obtained using a ZnSe window. Panels (B) and (C) correspond to expanded sections of the areas shown in green and blue, respectively, in panel (A). (D) Different view angles of the spatial mapping of the Co_3_O_4_/GO-CL amide band, measured with ATR FT-IR microscopy, within an area of 10 μm × 10 μm, along with the optical image of the sample. (E) High-resolution XPS spectrum of Co_3_O_4_/GO-CL with a peak fitting for N 1s.

The indications of covalent bonds in Co_3_O_4_/GO-CL observed with Raman and XRD were investigated through FTIR analysis. The top curve in Figure 3A exhibits the FTIR spectrum of pure Co_3_O_4_, where two strong bands at 659 and 558 cm^–1^ are visible and refer to vibration modes of Co–O in Co_3_O_4_ spinel for Co^2+^ and Co^3+^, respectively.^35^ The lower curve in Figure 3A shows the spectra of pure GO that, as a result of its highly oxygenated nature, presents a greater number of bands. It is possible to observe intense bands at 1736 and 1620 cm^–1^, related to carboxylic νC=O and δO–H of water molecules, respectively.^40^ Possibly, the 1620 cm^–1^ band is hindering observation of the band referred to as νC=C that would be expected to appear at 1570–1585 cm^–1^.^40^ Finally, the bands at 1390, 1246, 1109, and 864 cm^–1^ are referenced to δC–OH (carboxy), νC–O–C (epoxy), νC–O (alkoxy) and δC–O–C (epoxy).^39,41^

When Co_3_O_4_ is functionalized with APTES, a distinct spectrum can be observed. It is possible to note the appearance of bands at 2820 and 2900 cm^–1^, which are respectively related to asymmetric and symmetric νCH_2_ of propyl chains of APTES structure,^42^ and at 1120 and 1040 cm^–1^, referring respectively to νSi–O–Si and νSi–O–C.^42^ Besides that, it is possible to see the δΝ–Η band of the pure Co_3_O_4_ functionalized with APTES at 1597 cm^–1^ (see more details in Figure 3B).^43,44^ Furthermore, the presence of characteristic bands of both APTES (νSi–O–Si and νSi–O–C) and Co_3_O_4_ (Co–O modes) are found in Co_3_O_4_/GO-CL spectrum, suggesting the formation of the hybrid material.

By comparing GO with the heterostructure in Figure 3B, it is possible to note that the relative intensity of the band at 1736 cm^–1^ (carboxylic νC=O) is decreased, as well as the band at 1645 cm^–1^ (amidic νC=O) appears.^39,45^ This may indicate the replacement of some carboxylic groups of the GO surface by amide ones when Co_3_O_4_/GO-CL is formed.

Figure 3C shows, in more detail, the differences observed when comparing Co_3_O_4_/GO-CL and Co_3_O_4_ spectra in the 500–800 cm^–1^ region of the spectra. Note the shift of the band located at 558 cm^–1^ to 563 cm^–1^, as well as its broadening. The greater width of this band for the heterostructure may be related to a combination of vibrational modes besides the typical Co–O, such as CoO(OH) and Co(OH)_2_.^32^ This would be in accordance with the functionalization of Co_3_O_4_ by APTES, which enriches its surface with oxygenated groups, as illustrated in Figure 1.

In order to verify the consistency and reproducibility of the amide bond in Co_3_O_4_/GO-CL, which represents the success in the production of the covalent heterostructure, an FT-IR spectral mapping of a 10 μm × 10 μm area was carried out using an ATR objective. Figure 3D presents the optical images of the sample in two different view angles, along with the respective color-coded three-dimensional maps for the intensity of the 1645 cm^–1^ band (see in more detail in Figure 3B). The color scale bar shows that while warmer colors (e.g., reddish) identify the detection of the highest intensities of the band, the colder colors (e.g., bluish) represent the absence of detection. Generally, the map attests to the homogeneous presence of the amide band throughout the analyzed area, since it is predominantly composed of warm colors. Thus, we may conclude that the covalently bonded heterostructure was not only successfully synthesized but also has good chemical bond spatial homogeneity.

It is important to highlight that the band assigned to δO–H, at 1620 cm^–1^, previously detected in the GO spectrum, cannot contribute to the 1645 cm^–1^ signal detected in Co_3_O_4_/GO-CL (Figure 3D). Indeed, Figure S1 shows the FTIR spectra of the materials in the entire infrared spectral range, from which it is possible to attest that no water molecules are detected after Co_3_O_4_/GO-CL formation, as the prominent water band at 3420 cm^–1^ (δO–H) disappears.

Finally, high-resolution XPS has been used to characterize Co_3_O_4_/GO-CL, seeking to further attest to the presence of the amide bonds. Figure 3E shows the peak fitting corresponding to the N 1s binding energy, with peaks attributed to NH_2_ (401.6 eV) and the amide bond (399.7 eV).^46,47^ It is interesting to note that the amide bonds correspond to 70.85% of the atomic percentage, thus indicating that the majority of NH_2_ groups of APTES were covalently linked to GO through the amide bonds. The XPS survey and other important high-resolution spectra (C 1s, O 1s, Co 2p, and Si 2p) are presented in Figure S2, along with Table S1, which exhibit the binding energies and corresponding chemical groups. The curve for O 1s also confirms the presence of the amide bonds through the peak at 533.4 eV.^47^ These results are very promising considering that amide bonds are highly stable (e.g., against hydrolysis)^48,49^ and thus could mean obtaining a material with improved resistance for future applications. In fact, many works have shown that the presence of these bonds may improve the stability of materials applied as supercapacitors,^50^ batteries,^51^ and electrocatalysts.^52^

**Figure 4:**
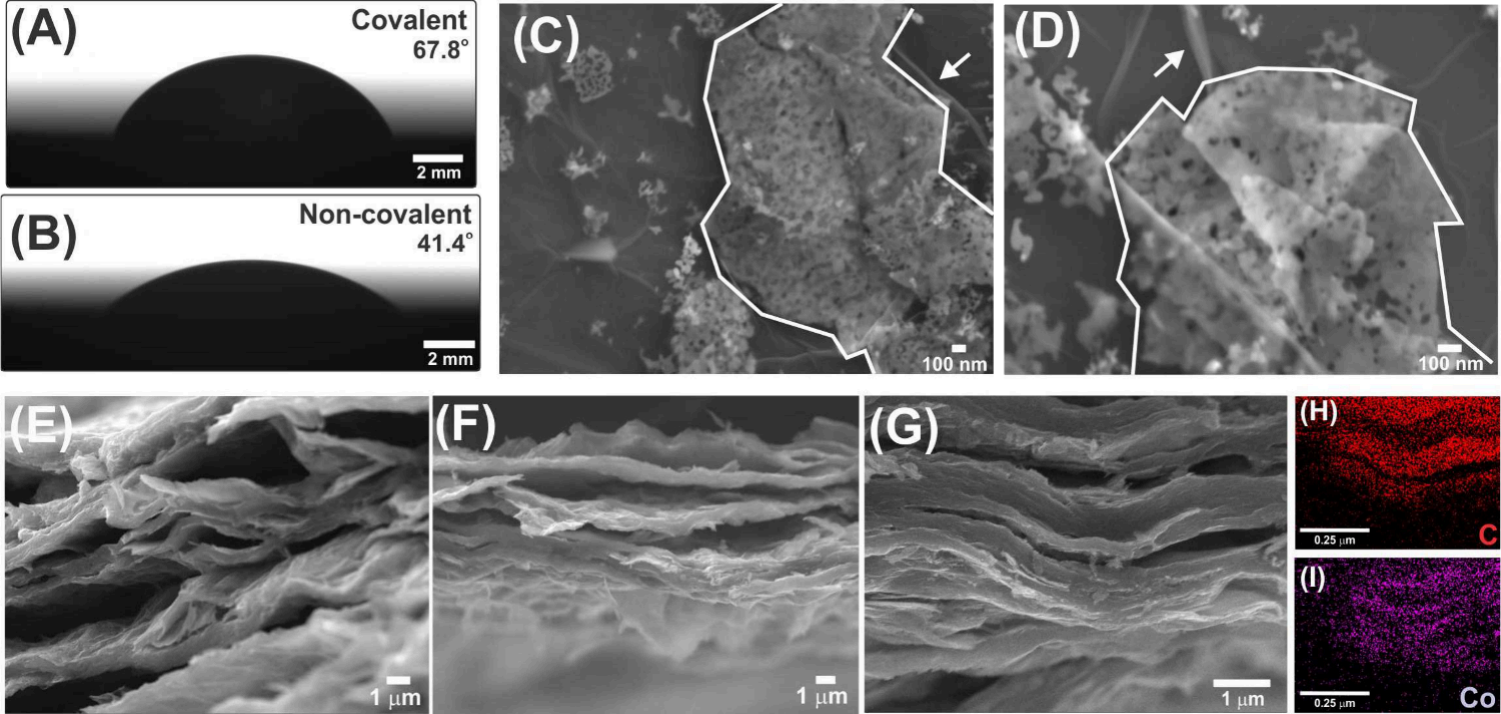
Optical images of a water droplet on the surface of (A) Co_3_O_4_/GO-CL and (B) Co_3_O_4_/GO-nCL heterostructures. SEM images of (C, D) Co_3_O_4_/GO-nCL and (E-G) Co_3_O_4_/GO-CL (cross-sectional). (H, I) EDS elemental mappings of Co_3_O_4_/GO-CL of panel (G).

The wetting characteristics of the heterostructures were investigated through water contact angle measurements, in which the liquid drop is placed on the material’s surface. The higher the contact angle between the liquid and the solid, the greater the hydrophobicity of the material. Figures 4A and 4B exhibit optical images of the drop on the heterostructures. Angle values of 67.8° ± 0.3° and 41.4° ± 2.2° were found for Co_3_O_4_/GO-CL and Co_3_O_4_/GO-nCL, respectively, indicating that the material with covalent bonds is more hydrophobic. In fact, a decrease in the material’s hydrophilicity would be expected after the functionalization due to some polar groups (e.g., carboxylic acids) present in the long APTES chains. On the other hand, Co_3_O_4_/GO-nCL still has its oxygen-containing functional groups available, thus implying lower contact angles.

Considering that morphology aspects could also enlighten the contact angle results, SEM analysis was carried out, and the obtained images are presented in Figures 4C–G, along with EDS elemental mappings of Co_3_O_4_/GO-CL in Figure 4H and 4I. SEM images that attest to the planar structure of 2D-Co_3_O_4_ are exhibited in the SI (see Figure S3).

Images of the noncovalent heterostructure (Figures 4C and 4D) show the typical wrinkled sheets of graphene-based materials (see arrows) and the highly porous sheets of Co_3_O_4_ (see straight line contours) randomly arranged one on top of the other. In the case of the Co_3_O_4_/GO-CL heterostructure (Figures 4E–G), however, the cross-sectional images show a very ordered pattern, inferring that the covalent bond between Co_3_O_4_ and GO induces parallel stacking and the formation of a layered structure. In fact, this corroborates the contact angle results, since the compact arrangement of Co_3_O_4_/GO-CL surely decreases the surface contact area accessible to water droplets^8^ and leads to high contact angles, as observed. Finally, EDS mappings (Figures 4H and 4I) attest to Co_3_O_4_/GO-CL’s composition by showing the homogeneous distribution of carbon and cobalt throughout the organized network.

In summary, remarkable differences between the covalent and noncovalent heterostructures could be noted, mainly related to the final architecture of the materials. The novelty in these findings motivated an investigation in order to propose a growth mechanism of Co_3_O_4_/GO-CL.

**Figure 5:**
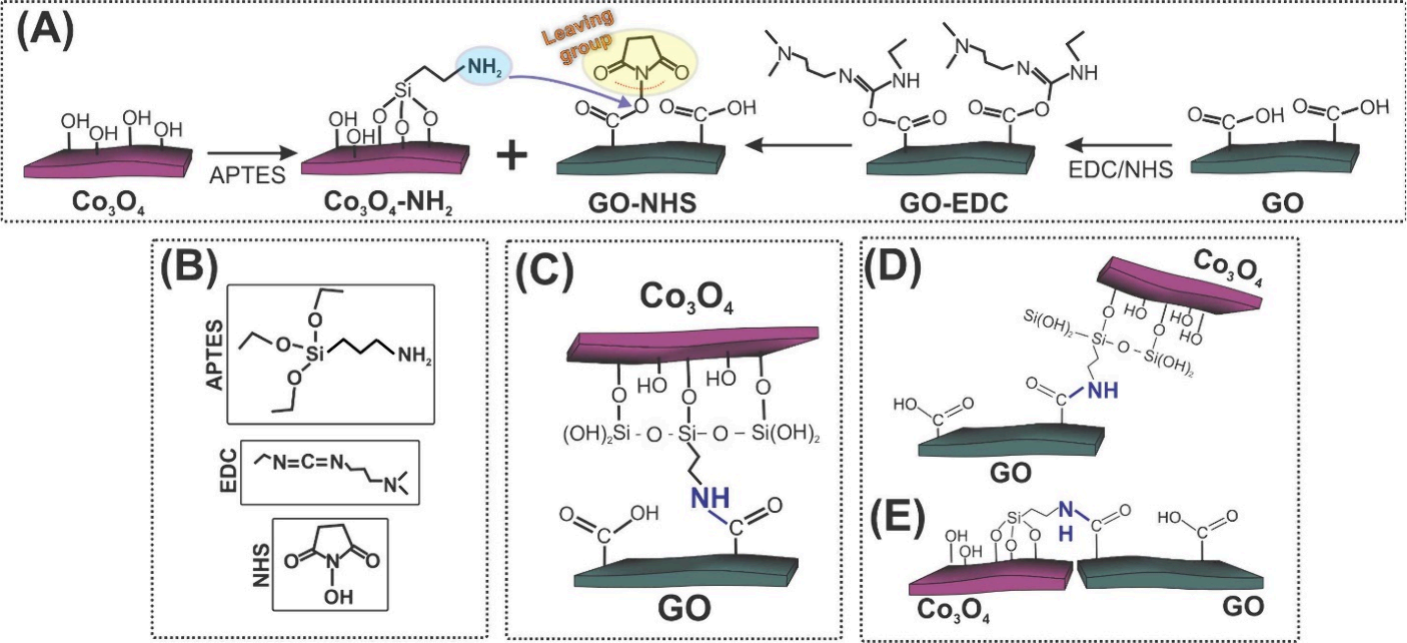
(A) Proposed reaction mechanism for the formation of Co_3_O_4_/GO-CL. Representations of the (B) molecules involved in the reaction, (C) main configuration, and (D, E) less-favorable configurations of Co_3_O_4_/GO-CL.

Figure 5A shows the proposed reaction mechanism for the production of Co_3_O_4_/GO-CL. As stated, APTES rapidly reacts with the available −OH groups of the 2D-Co_3_O_4_ surface,^27,28^ leaving a structure of free −NH_2_ groups (see Co_3_O_4_–NH_2_ in Figure 5A). Subsequently, the carboxylic groups of the GO surface are activated through the action of a coupling reagent (EDC/NHS), which converts the inactive carboxylic acid of GO to activated carboxylates. Thus, in the proposed reaction, the first step involves the reaction of the hydroxyl group of the GO carboxylic acid with the carbodiimide reagent (EDC), resulting in the O-acylurea derivative (see GO-EDC). This intermediate then undergoes a nucleophilic attack by NHS, forming the succinimidyl ester (−COOSuc), represented by GO-NHS in Figure 5A, which is stable (i.e., a good leaving group) and allows the efficient replacement by primary amines,^53^ such as −NH_2_ of APTES. Molecules are depicted in Figure 5B.

The main configuration observed throughout the characterization of Co_3_O_4_/GO-CL is depicted in Figures 4E–G and showed a lamellar organization among the layers, such as that represented in Figure 5C. However, it is possible that other less-favorable structures are formed, mainly if we consider that the carboxylic groups are primarily placed on the edges of GO layers.^54^ In fact, SEM images of Co_3_O_4_/GO-CL (see Figure S4) showed specific regions, which indicates that these connections may also happen (i) through the bond of the Co_3_O_4_ basal plane with the border of GO (Figures 5D and S4A), and (ii) through a side-by-side connection (Figures 5E and S4B).

To understand the impact of this diversity, as well as to infer potential applications, the properties of these materials were assessed through catalytic and electrochemical measurements.

### Materials Applications

#### Catalytic Degradation of Rhodamine 6G

**Figure 6:**
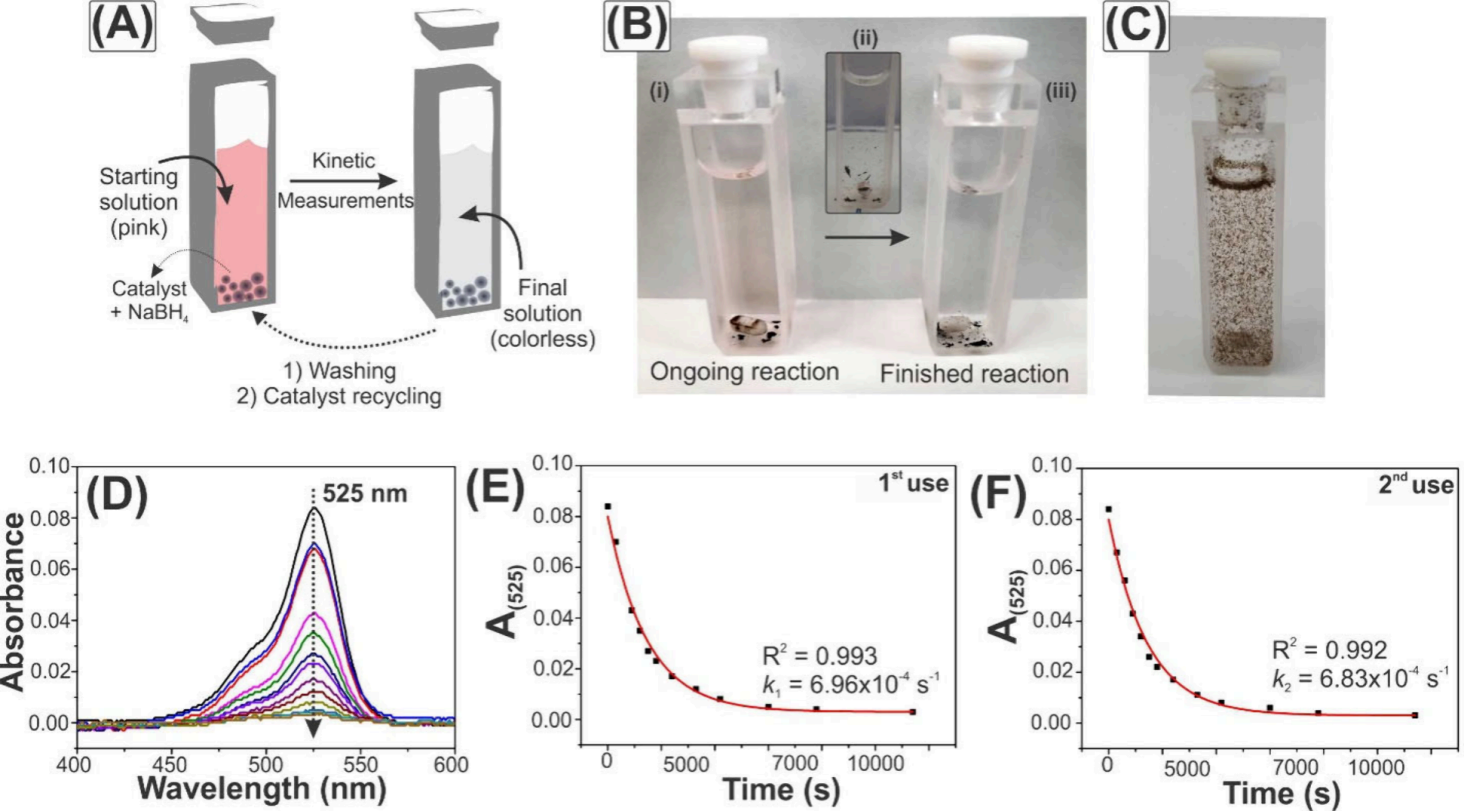
(A) Illustrative scheme of the catalysis. Pictures of the (B) reaction medium before and after catalysis with Co_3_O_4_/GO-CL and (C) reaction medium with Co_3_O_4_/GO-nCL. (D) UV-vis spectra acquired over time with the evolution of the band at 525 nm; and (E, F) kinetic profiles obtained after the first use (panel (E)) and second use (panel (F)) of Co_3_O_4_/GO-CL.

The catalytic reactions for the degradation of R6G were followed spectrophotometrically (at 525 nm) by monitoring the disappearance of the dye’s signal, which leads to a color change in the solution (from pink to colorless), as illustrated in Figure 6A. As mentioned in the Experimental Section, kinetic measurements were carried out periodically, by stopping the magnetic stirring and taking the cuvette to the spectrophotometer. Figures 6B and 6C show digital photographs of the cuvettes containing Co_3_O_4_/GO-CL and Co_3_O_4_/GO-nCL, respectively, in the dye solution. The reaction process of the cross-linked heterostructure clearly shows that the solid originally decanted on the bottom of the cuvette (Figure 6B(i)) is agitated when magnetic stirring is turned on (Figure 6B(ii)) and then, after ceasing agitation, returns to the initial decanted state (Figure 6B(iii)). This allows the solution to be easily measured with a spectrophotometer once the solid separates quickly, ensuring that there is no interference in the analysis. This behavior, on the other hand, is not observed for the noncovalent hybrid. Figure 6C shows that the material spreads throughout the solution with or without stirring and, after some time, it starts to disperse, indicating that the layers are not as strongly connected as in Co_3_O_4_/GO-CL. This could impair the monitoring of the reaction by UV-vis, since there will be interference from species other than R6G. Figure S5 shows that (i) R6G and Co_3_O_4_ appear in the same range (Figure S5A) and (ii) after adding the catalyst and the reduction agent, the band of Co_3_O_4_ seems to interfere in the spectrum (Figure S5B). In this sense, the reaction under the catalytic actuation of Co_3_O_4_/GO-nCL could not be monitored and we shall focus on the performance of Co_3_O_4_/GO-CL.

Figure 6D shows the band of R6G at 525 nm that was monitored and that is related to the delocalization of electrons in the amino N atom to the positively charged iminium site.^55^ The decrease in its absorbance with time indicates the degradation of the dye. In order to infer the possibility of reusing the catalyst, two consecutive reactions were monitored by simply recovering the powder after its first use, washing it with a filtration process, drying (60 °C), and reusing it in a new R6G solution. In fact, this characterizes the reaction as a heterogeneous catalysis, i.e., catalysts that are not in the same phase as the analyte, which is very advantageous as it facilitates the catalyst recovery.

The absorbance versus time data obtained are depicted in Figures 6E and 6F, as well as the kinetic profile fittings for the degradation process, which correspond to pseudo-first-order reaction curves, with respect to the concentration of R6G (see eq S1).^56^ Rate constants of 6.96 × 10^–4^ s^–1^ and 6.83 × 10^–4^ s^–1^ were found and are comparable to rates in the literature, on the order of 10^–3^ and 10^–4^ s^–1^.^57,58^ Besides that, they clearly show that the catalytic activity of Co_3_O_4_/GO-CL is maintained, suggesting great stability and the possibility of recycling the catalyst.

For comparison, the degradation reaction was also followed in the absence of Co_3_O_4_/GO-CL. Figure S6 shows that R6G is not completely degraded, even after long monitoring periods, attesting to the role of Co_3_O_4_/GO-CL as a catalyst in R6G degradation.

Overall, we propose that R6G and the reducing agent NaBH_4_ are adsorbed on the Co_3_O_4_/GO-CL lamellar structure, which contribute to faster electron transfer processes from the donor (BH_4_^–^) to the acceptor (R6G),^55,58^ leading to its reduction and further degradation. Indeed, the ordered cross-linked sheets of Co_3_O_4_/GO-CL may ensure enhanced interfacial area and charge-transfer between the layers, as reported for other covalently bonded heterostructures,^13^ possibly favoring its catalytic performance.

#### Electrochemical Evaluation

Before starting the electrochemical experiments, in order to ensure a high sheet conductivity for the carbonaceous material, it underwent a reduction procedure with hydrazine vapor,^59^ leading to Co_3_O_4_/rGO-CL and Co_3_O_4_/rGO-nCL. Characterization of these samples was carried out by Raman and the results are depicted in Figure S7, in comparinson with the nonreduced materials. Two main differences may be observed when comparing the structures that went through the reduction process and those that did not. Regarding the G and D bands related to graphene derivatives, it is possible to note an increase in the intensity of the D band in comparison to the G band, in both cases (covalent and noncovalent materials), which is in accordance with the reduction of GO.^33^ Besides that, a blue shift is detected in the A_1g_ bands of Co_3_O_4_ that may related to an increase in the oxygen vacancies on the surface of the material.^60^

**Figure 7:**
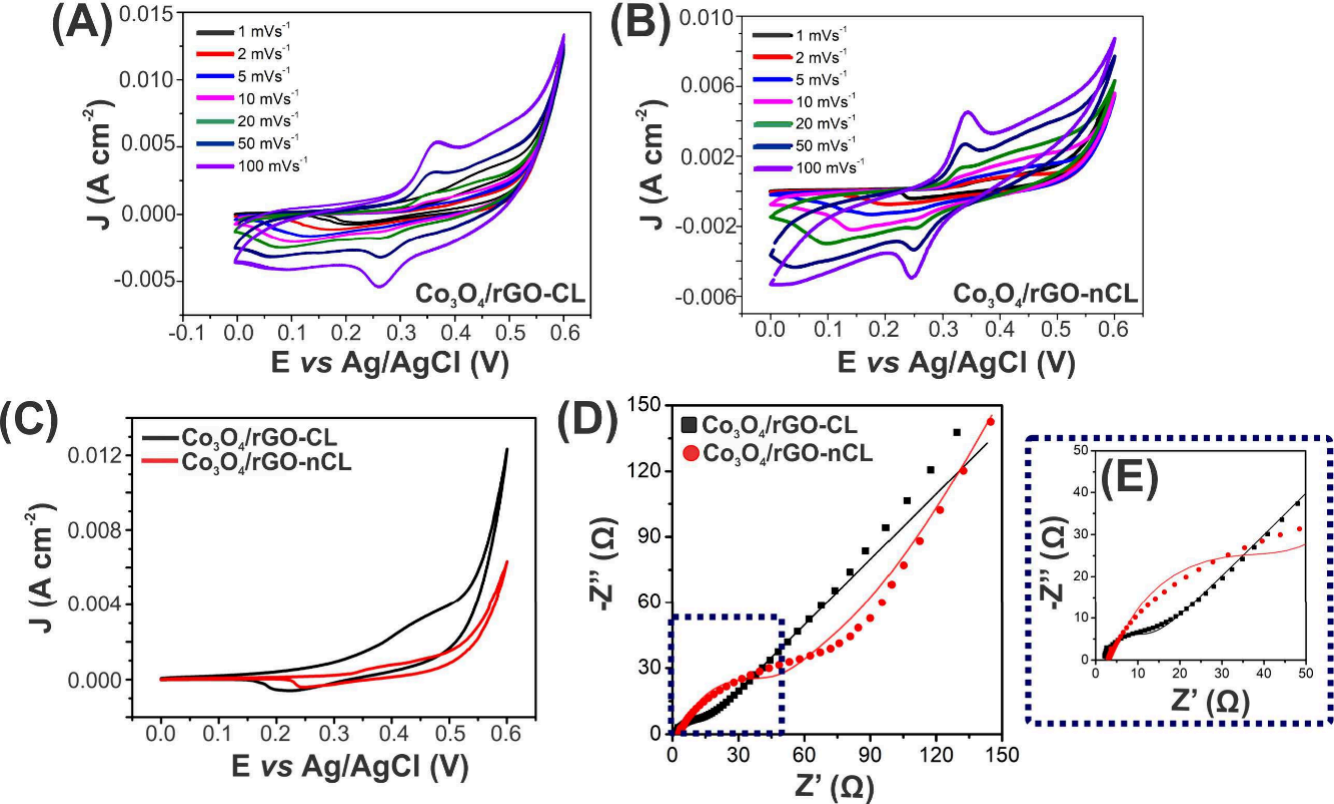
Cyclic voltammograms (CVs) of (A) Co_3_O_4_/rGO-CL and (B) Co_3_O_4_/rGO-nCL at different scan rates; (C) comparison between CVs at 1 mV s^–1^ of both materials; and (D, E) fitted Nyquist plots of Co_3_O_4_/rGO-CL and Co_3_O_4_/rGO-nCL, with 2 M KOH electrolyte.

Since we are interested in investigating the electrochemical characteristics of the electrode active hybrids, and not in the construction of a supercapacitor device,^61−63^ we have systematically characterized the materials in a three-electrode system. Results of cyclic voltammogram (CV) and electrochemical impedance spectroscopy (EIS) in 2 M KOH are shown in Figure 7.

Figures 7A and 7B show the CVs in the range of 0.0–0.6 V at scan rates from 1 mV s^–1^ to 100 mV s^–1^ for both the covalent and noncovalent structures. The nonrectangular curves exhibit two pairs of asymmetric redox peaks related to the formation of multiple cobalt oxide phases at different oxidation states (Co^2+^ ↔ Co^3+^ ↔ Co^4+^)^64^ and reveal the pseudocapative feature of Co_3_O_4_.^65^ It is possible to note that the current density increases as the scan rate gets higher, and the oxidation and reduction peaks shift to higher and lower potentials, respectively. This is expected, due to the different diffusion of ions toward the material under the different conditions.^66^ Finally, by comparing the profiles in Figure 7C, an increase in the current density can be observed from of Co_3_O_4_/rGO-nCL to Co_3_O_4_/rGO-CL. This would be in accordance with the open geometry that the covalent heterostructure presents (see Figure 4), which possibly provides rich active sites for electrolyte penetration.^67^ Moreover, the covalent bonds may facilitate axial charge transfer.^68^ Thus, in order to investigate the electrochemical properties of the materials in energy storage, we have estimated their specific capacitance (*C*_sp_) by integrating the CV curves under 1 mV s^–1^, following eq S2.^65^ Values of *C*_sp_ for Co_3_O_4_/rGO-nCL and Co_3_O_4_/rGO-CL were found to be 110 F g^–1^ and 468 F g^–1^, respectively. This result underlines the role of the covalent bonds in the structure and attests to the possibility of producing a more efficient electrode by following this approach, as previously reported for other materials.^67−69^

Finally, EIS analysis has been performed aiming at probing information on charge transfer resistance and diffusion kinetics of electrons on the electrode surface. The Nyquist plots presented in Figures 7D and 7E may be basically divided into two parts: (i) a high-frequency region that contains a semicircle that represents the electron transfer impedance (detailed in Figure 7E) and (ii) a low-frequency region with an inclined line that is related to the ion diffusion impedance.^70^ In the first range, the intercept of the semicircle with the real axis (*Z*') gives information about the electrolyte resistance (*R*_e_), and its diameter implies the charge-transfer resistance (*R*_ct_). In the second range, the line slope dictates how the diffusion process happens.^71^Figure S8 shows the circuits used to fit the curves.

By comparing the curves of Co_3_O_4_/rGO-nCL and Co_3_O_4_/rGO-CL it is noticeable that the semicircle and the intercept of the covalently linked structure (black) are much smaller than the noncovalent (red), indicating an improved kinetics of charge transfer for this material. In fact, *R*_ct_ values were 26.5 and 9.07 Ω for Co_3_O_4_/rGO-nCL and Co_3_O_4_/rGO-CL, respectively. Regarding the inclined lines, the larger slope observed for the cross-linked heterostructure suggests faster ion diffusion.^72^ Thus, it demonstrates that the covalent bonds may contribute to the regulation of the charge-transfer transport and reduction of ion-diffusion barriers.

## Conclusions

In summary, we have demonstrated that it is possible to covalently connect 2D-Co_3_O_4_ and GO layers for the generation of Co_3_O_4_/GO heterostructures via a simple route. By comparing it with its noncovalent analogous, different morphological, structural, and physicochemical properties were identified. Catalytic studies with Co_3_O_4_/GO-CL, for the degradation of R6G, led to rate constants of 6.96 × 10^–4^ s^–1^ and 6.83 × 10^–4^ s^–1^ in different reaction cycles, which were carried out by recovering and reusing the catalyst, therefore attesting to the great stability and performance of Co_3_O_4_/GO-CL. Co_3_O_4_/GO-nCL, on the other hand, suffered from critical deterioration during catalysis, not allowing the reaction to be monitored. Electrochemical activity of Co_3_O_4_/GO-CL was noteworthy with 5 times increased capacity of store energy compared to Co_3_O_4_/GO-nCL: 468 F g^–1^ against 110 F g^–1^. EIS results showed *R*_ct_ values of 26.5 and 9.07 Ω for Co_3_O_4_/rGO-nCL and Co_3_O_4_/rGO-CL, respectively. This indicated that the covalent bonds allow faster ion-diffusion and charge-transfer kinetics, leading to improved Co_3_O_4_/GO-CL capabilities. In this sense, we have been able to show that heterostructures of 2D-Co_3_O_4_ and GO, when covalently linked, may exhibit novel properties not observed in hybrids prepared through conventional experimental methods. Besides that, this work offers an insight toward the intriguing confined and controlled spaces between covalently linked layers, which has been shown to favor different chemical processes that are worthy of being investigated. These new concepts enlighten new possibilities toward the architecture of 3D platforms based on covalently bonded 2D layer stacks.

## Supporting Information Available

Additional experimental details. SEM images, XPS data, spectra of UV-vis and Raman characterizations; kinetic and specific capacitance equations (PDF)
