# Supplementary material for: Covalently Linked 2D-Co3O4/GO Heterostructures: Catalytic and Electrochemical Properties
Source: Langmuir. 2024 Oct 3;40(41):21442–52. doi: 10.1021/acs.langmuir.4c02235 (PMC11483772; doi:10.1021/acs.langmuir.4c02235)
Supplement: Supplementary file 1 — la4c02235_si_001.pdf [file la4c02235_si_001.pdf]

SUPPORTING INFORMATION

Covalently linked 2D-Co_3O_4/GO heterostructures: catalytic and electrochemical properties

*Jéssica E. S. Fonsaca,†‡ Carlos Eduardo Lima,†‡ Kevin Stefan Boszko Martins,†‡ Sergio H. Domingues,†‡ Christiano J. S. de Matos†‡ * christiano.matos@mackenzie.br*

†School of Engineering, Mackenzie Presbyterian University, Sao Paulo – 01302-907, Brazil.

‡MackGraphe, Mackenzie Presbyterian Institute, Sao Paulo – 01302-907, Brazil.

Table of Contents

Experimental procedure S3

Synthesis of Graphene Oxide (GO)..S3

Synthesis of Co_3O_4..S3

FTIR spectra of Co_3O_4/GO-CL and GO..S5

Figure S1. FTIR spectra of Co_3O_4/GO-CL and GO. S5

XPS spectra of Co_3O_4/GO-CL..S6

Figure S2. (A) XPS survey spectrum and (B-E) fitted high-resolution XPS spectra of (B) C 1s, (C) O 1s, (D) Co 2p and (E) Si 2p of Co_3O_4/GO-CL. S6

Table S1. Binding energy values obtained from high resolution XPS spectra for C 1s, O 1s, Co 2p and Si 2p of Co_3O_4/GO-CL..S6

SEM images of 2D-Co_3O_4..S9

Figure S3. (A-D) SEM images of 2D-Co_3O_4. S9

SEM images of Co_3O_4/GO-CL S10

Figure S4. (A-B) SEM images of Co_3O_4/GO-CL. S10

UV-Vis spectra of pure R6G, Co_3O_4 and GO S11

Figure S5. (A) UV-Vis spectra of R6G solution (red) and dispersions of Co_3O_4 (blue) and GO (black). (B) UV-Vis spectra of pure R6G (blue) and after adding the catalyst and the reduction agent (red and black). S11

Kinetic parameters S12

Profiles of absorbance *versus* time: comparing with the control reaction S13

Figure S6. Absorbance of the band at 525 nm versus time comparing the reaction in the absence (blank) and the presence of catalyst. S13

Raman spectra of materials after reduction S14

Figure S7. Raman spectra of heterostructures before (Co_3O_4/GO-nCL and Co_3O_4/GO-CL) and after (Co_3O_4/rGO-nCL and Co_3O_4/rGO-CL) reduction with vapor of hydrazine. S14

Specific capacitance calculation S15

Equivalent circuit of Nyquist Plot for Co_3O_4/GO-CL and Co_3O_4/GO-nCL S16

Figure S8. Equivalent circuit of Nyquist Plot for (A) Co_3O_4/GO-CL and (B) Co_3O_4/GO-nCL. S16

Experimental procedure

Synthesis of Graphene Oxide (GO)

Graphene Oxide (GO) was synthesized by a modified Hummer's method. For the oxidative process, a beaker containing 1.0 g of graphite (Nacional do Grafite - Graflake 95580) and 60.0 ml of sulfuric acid (Sigma Aldrich) was kept under magnetic stirring (2000 rpm) for 15 minutes. The system was then transferred to an ice bath, where 3.5 g of KMnO4 (Sigma-Aldrich) was slowly added in a total time of 120 minutes. After this addition, the ice bath was removed, and the system was kept stirring for 3 days. Then, 150.0 mL of deionized water and 3.0 mL of a H_2O_2 solution (30% v/v) were added to interrupt the reaction, and the system was ready for washing. A 10% (v/v) solution of HCl was added and kept stirring for 1h and resting for 24h for decantation. After that, the supernatant was removed, and the process was repeated for more 2 times: one with HCl 10% (v/v) and one with deionized water. Finally, the product was sonicated in ultrasonic bath for 15 minutes, leading to the GO dispersion.

Synthesis of Co_3O_4

In a round flask, 16.25 mL of ethanol and 1.00 mL of deionized water were added, followed by the reagents, cobalt acetate tetrahydrate ($(CH_3COO)_2Co.4H_2O$) and hexamethylenetetramine

($C_6H_{12}N_4$), in the quantities of 4.2×10^{-4} mol and 4.9×10^{-4} mol, respectively. This mixture was kept stirring (1000 rpm) for 15 minutes under room temperature until the complete dilution of the reagents. Next, the final solution was added to a 50 mL Teflon-lined stainless-steel autoclave and taken to a furnace set to 170°C, where it was maintained for 2h. After this period, the precipitates resultant from the reaction were washed with deionized water and ethanol through centrifugation (4000 rpm) for at least three cycles of 15 min with each solvent. The material was dried at 50°C (12h) and taken to the final step: heat treatment at 450°C for 3h, leading to Co_3O_4 as a powder.

FTIR spectra of Co_3O_4/GO-CL and GO

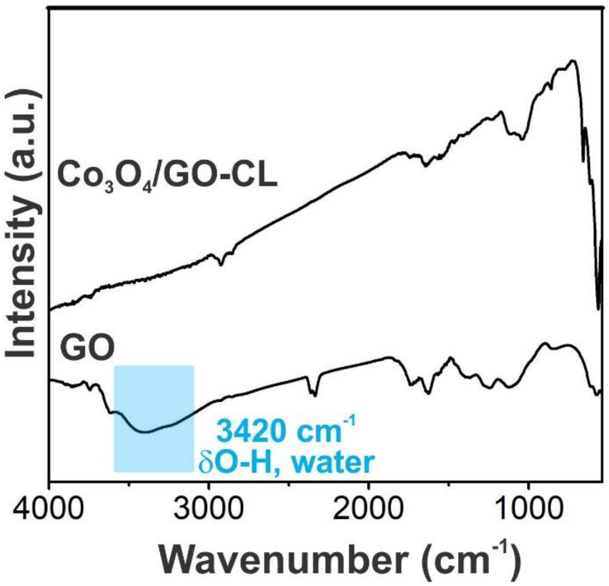

Figure S1. FTIR spectra of Co_3O_4/GO-CL and GO.

XPS spectra of Co_3O_4/GO-CL

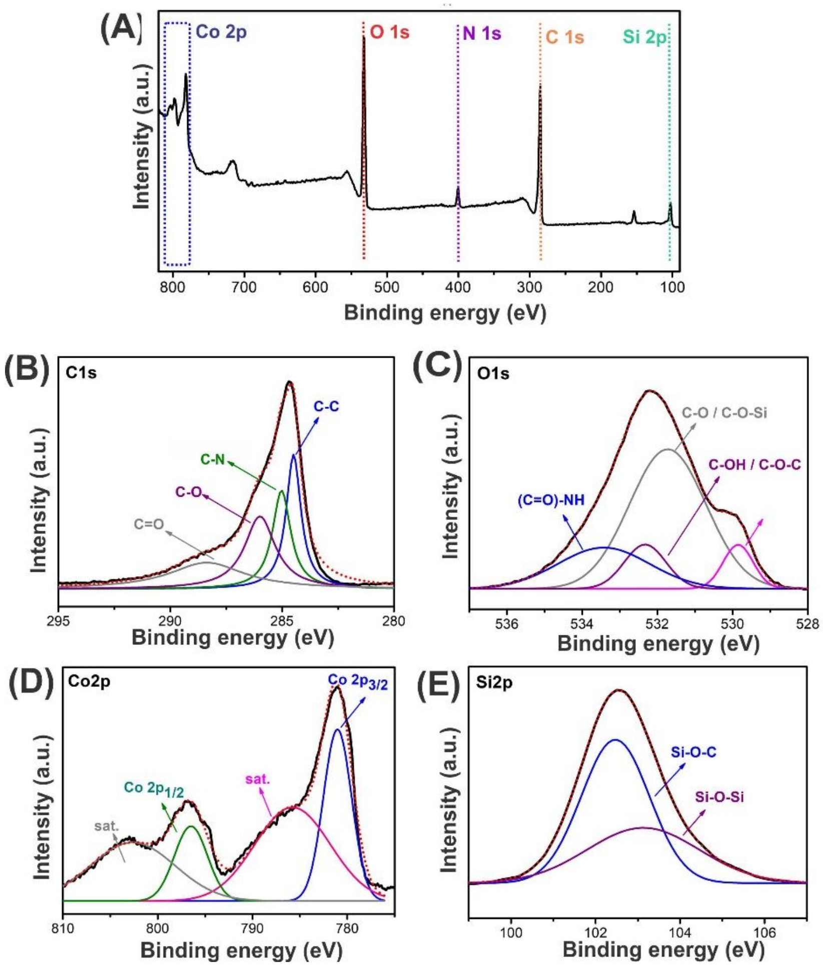

Figure S2. (A) XPS survey spectrum and (B-E) fitted high-resolution XPS spectra of (B) C 1s, (C) O 1s, (D) Co 2p and (E) Si 2p of Co_3O_4/GO-CL.

Table S1. Binding energy values obtained from high resolution XPS spectra for C 1s, O 1s, Co 2p and Si 2p of Co_3O_4/GO-CL.

	Chemical group	Binding energy (eV)	Ref
	C-C	284.4	[1]
	C-N	285.5	[1]

**C 1s**	C-O	286.1	[1]
	C=O	288.0	[1]
**O 1s**	O-C=O	529.9	[2]
	C-O/C-O-Si	531.8	[2]
	C-OH / C-O-C	532.3	[3]
	(C=O)-NH, amide	533.4	[3]
**Co 2p**	Co $2p_{3/2}$	781.0	[4]
	Satellite	788.0	[4]
	Co $2p_{1/2}$	796.4	[4]
	Satellite	802.0	[4]
**Si 2p**	Si-O-C	102.4	[5]
	Si-O-Si	103.1	[5]

REFERENCES:

[1] Domingues, S. H.; Kholmanov, I. N.; Kim, T.; Kim, J.; Tan, C.; Chou, H.; Alieva, Z. A.; Piner, R.; Zarbin, A. J. G.; Ruoff, R. S. Reduction of graphene oxide films on Al foil for hybrid transparent conductive film applications. *Carbon* 2013, *63* (0), 454-459.

[2] Lee, J. H.; Kim, S. H. Fabrication of silane-grafted graphene oxide and its effect on the structural, thermal, mechanical, and hysteretic behavior of polyurethane. *Sci. Rep.* **2020**, *10* (1), 19152.

[3] Santos, Y. H.; Martinez, A. H. G.; Veiga, A. G.; Rocco, M. L. M.; Zarbin, A. J. G.; Orth, E. S. Neighboring Effects on the Selective Bifunctionalization of Graphene Oxide for Nanocatalytic Organophosphate Neutralization. *ACS Applied Nano Materials* **2022**, *5* (5), 6001-6012.

[4] M.A. Ehsan, A.S. Hakeem, A. Rehman, Hierarchical Growth of CoO Nanoflower Thin Films Influencing the Electrocatalytic Oxygen Evolution Reaction, Electrocatalysis, 11 (2020) 282-291.

[5] Ramirez-Soria, E. H.; León-Silva, U.; Lara-Ceniceros, T. E.; Bazán-Díaz, L.; Advíncula, R. C.; Bonilla-Cruz, J. Graphene oxide bifunctionalized with NH2/NH3+ and their outstanding-performance against corrosion. *Applied Surface Science* **2021**, *561*, 150048.

SEM images of 2D-Co_3O_4

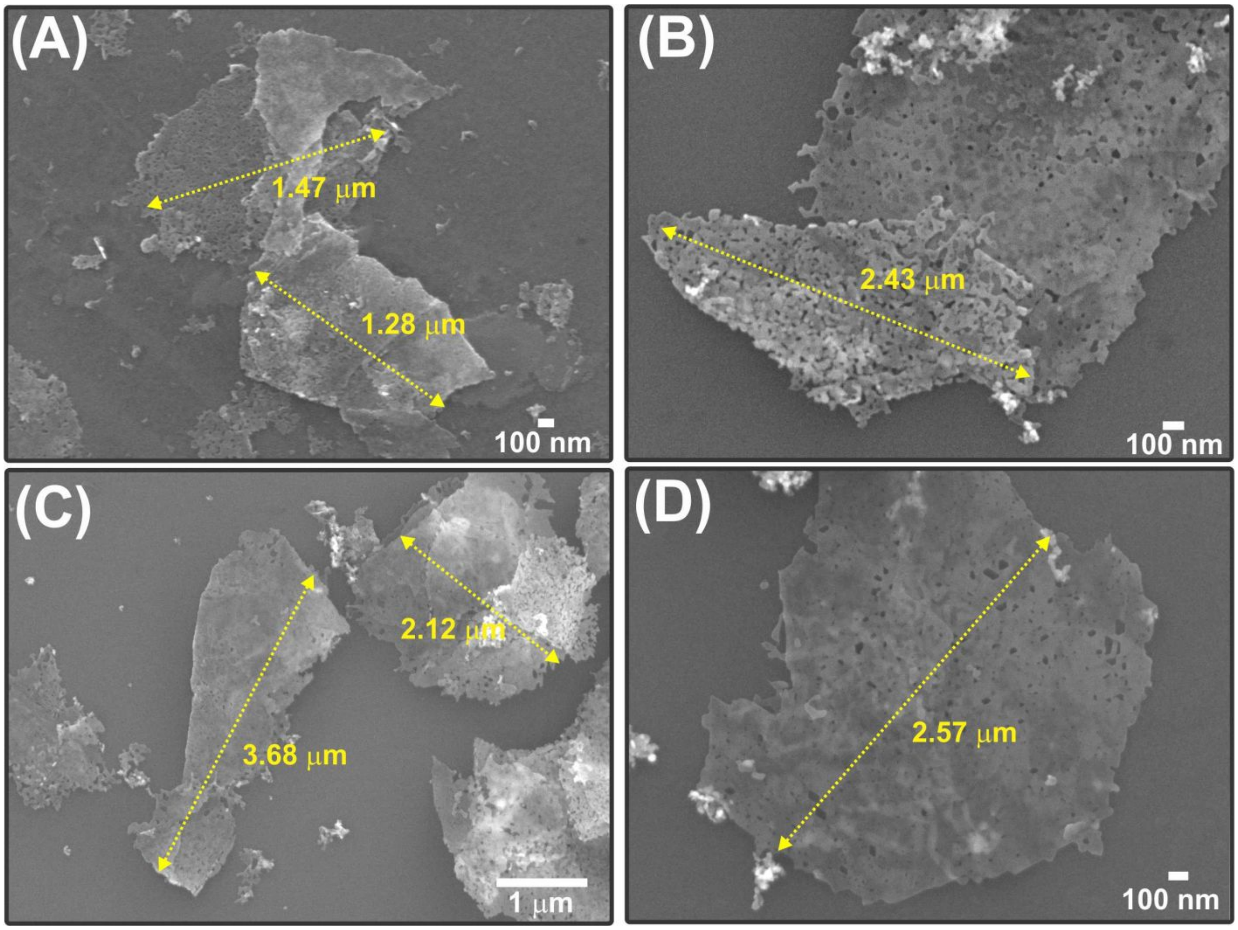

Figure S3. (A-D) SEM images of 2D-Co_3O_4.

SEM images of Co_3O_4/GO-CL

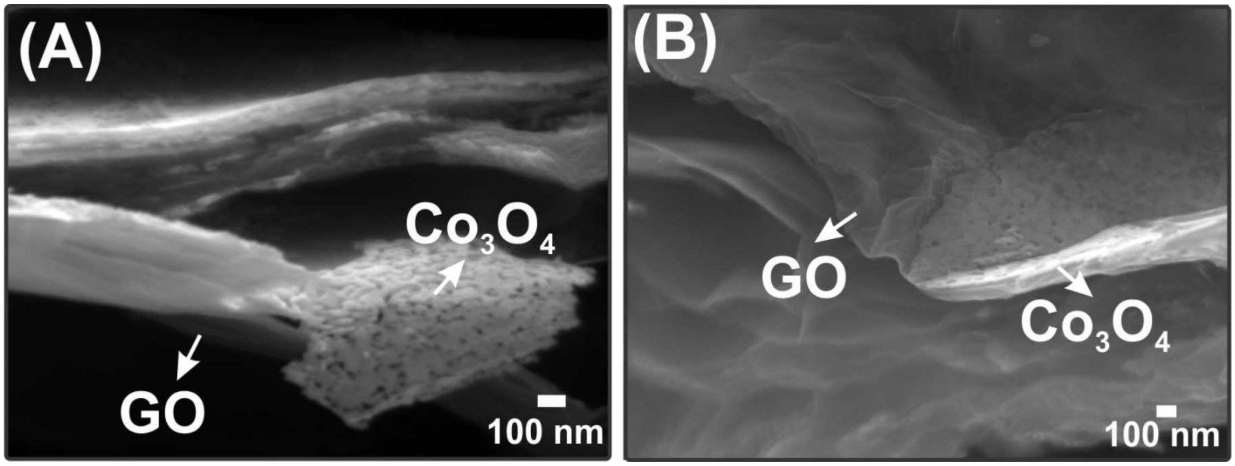

Figure S4. (A-B) SEM images of Co_3O_4/GO-CL.

UV-Vis spectra of pure R6G, Co_3O_4 and GO

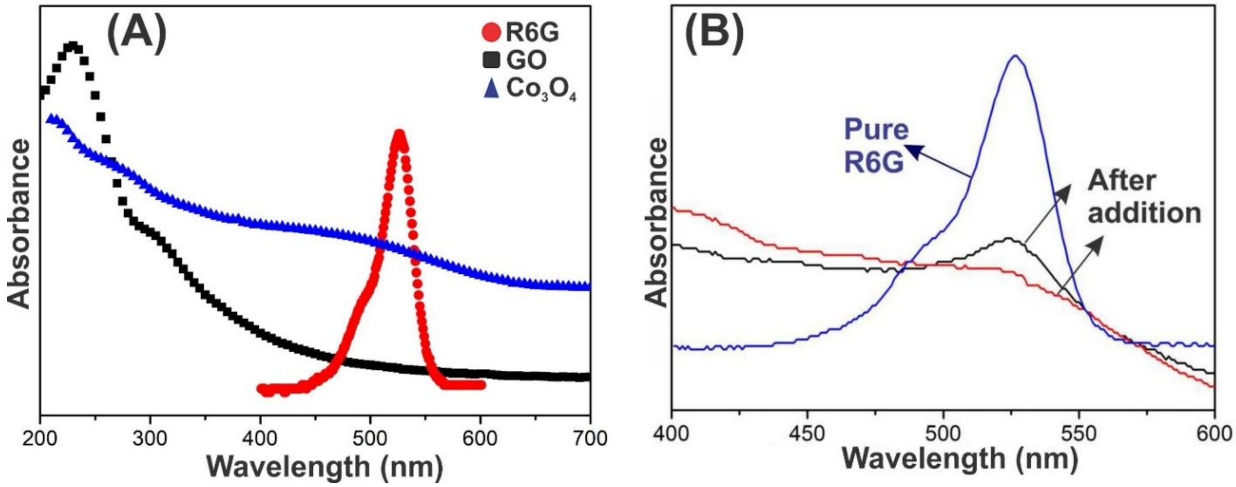

Figure S5. (A) UV-Vis spectra of R6G solution (red) and dispersions of Co_3O_4 (blue) and GO (black). (B) UV-Vis spectra of pure R6G (blue) and after adding the catalyst and the reduction agent (red and black).

Kinetic parameters

Equation S1:

$$A_t = (A_0\text{-}A_f) \times (\exp(-k_1 t)) + A_f$$

Where:

A_f = final absorbance of reagent degradation

A_0 = initial absorbance of reagent degradation

k_1 = kinetic constant of R6G degradation

Profiles of absorbance *versus* time: comparing with the control reaction

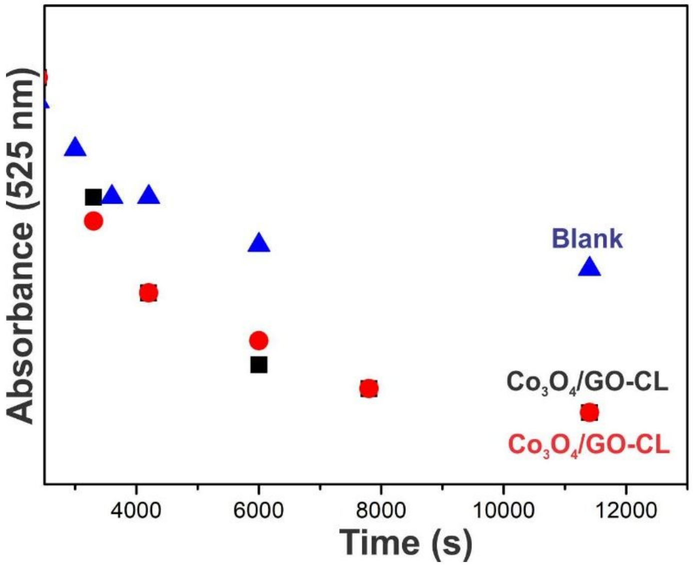

Figure S6. Absorbance of the band at 525 nm *versus* time comparing the reaction in the absence (blank) and the presence of catalyst.

Raman spectra of materials after reduction

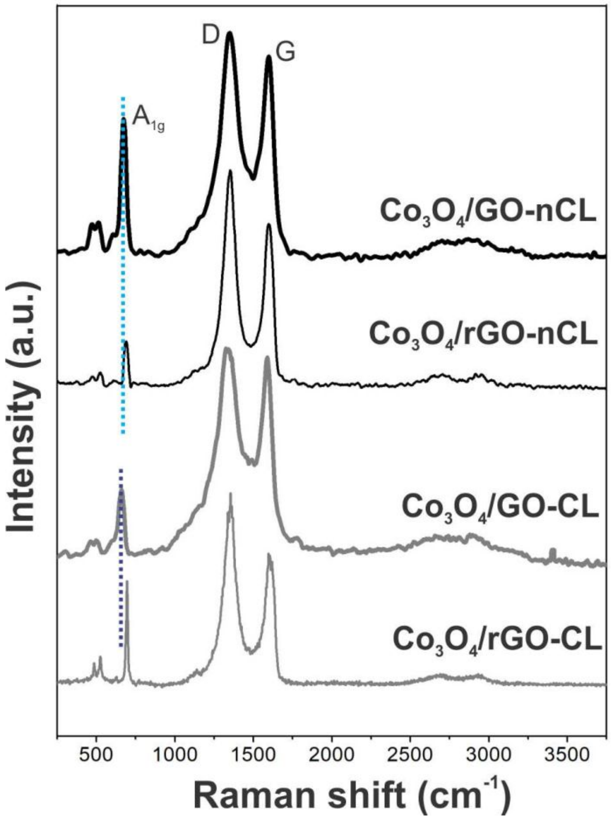

Figure S7. Raman spectra of heterostructures before (Co_3O_4/GO-nCL and Co_3O_4/GO-CL) and after (Co_3O_4/rGO-nCL and Co_3O_4/rGO-CL) reduction with vapor of hydrazine.

Specific capacitance calculation

Equation S2:

$$Csp = \frac{1}{\upsilon\, m\, \Delta V} \int I\, dV$$

Where *Csp* is the specific capacitance (F g^{-1}), *I* is the voltammetry current (A), υ is the scan rate (V s^{-1}), *m* is the mass of the active materials (g) and ΔV is the potential (V).

Equivalent circuit of Nyquist Plot for Co_3O_4/GO-CL and Co_3O_4/GO-nCL

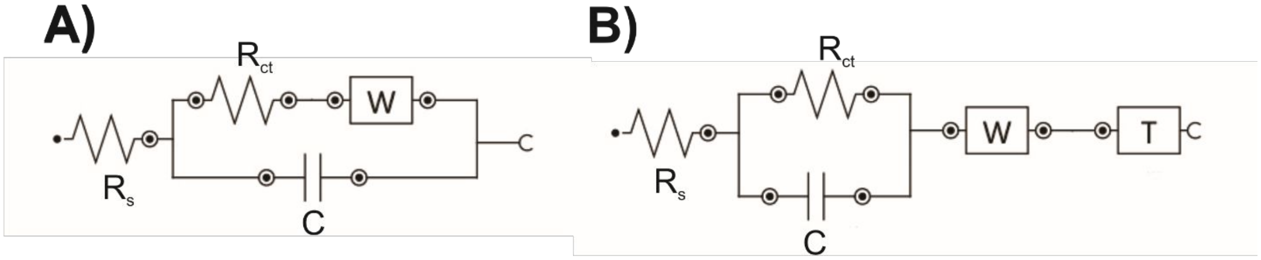

Figure S8. Equivalent circuit of Nyquist Plot for (A) Co_3O_4/GO-CL and (B) Co_3O_4/GO-nCL.

Where R_s is the bulk solution resistance, R_{ct} is the ion charge transfer resistance, C capacitance, W is the Warburg impedance and T is the Warburg impedance open.
